# A Few Remarks upon Aachen (Aix-La-Chapelle)

**Published:** 1885-06

**Authors:** E. P. Philpots


### A FEW REMARKS UPON AACHEN (AIX-LA-CHAPELLE).

BY

E. P. PHILPOTS, M.D., BOURNEMOUTH.

---

HAVING made several short stays at Aachen whilst *en route* for the Rhine, opportunities were afforded me of inspecting the mineral springs and baths, and I was thus led to form a favourable opinion of them as remedies for rheumatism, as also to send several rheumatic patients there, most of whom derived benefit by going. Having broken down from a severe attack of lumbago and sciatica this year, I tried the effects of these waters upon myself, and thinking that there might be various points connected with my visit which would interest some members of my profession, I have thought well to write a short paper thereon. The waters contain chloride, bromide, iodide, and sulphide of sodium, the former in abundance; the sulphates of soda and potash, and the carbonates of soda, lithia, magnesia, lime, strontia, and iron; also an appreciable amount of free carbonic acid. They rise at a temperature of 135°, and have a mawkish sulphurous taste. I derived great benefit from their use; I drank them in large quantities, and bathed in them twice a day, the bath temperature being about 104°, and each immersion lasting about half an hour. As it is well known that Aachen has obtained a very great and just notoriety as a resort for patients suffering from secondary and tertiary syphilis, who come there from all parts of the world for "the cure," it may interest some of the readers of this paper to be told something concerning this "cure." Possibly the waters aid it; but this is a doubtful point—at least, many persons think so. After the patients have been for half an hour in the sulphurous water, at a temperature of 95°, they are dried and rubbed with mercurial ointment for twenty minutes, professional rubbers being employed; about three drams of the ointment are used at one rubbing, and from twenty to eighty daily rubbings undergone, according to circumstances. These rubbings are usually commenced over the solei muscles; the next day the thighs are rubbed; then follow the abdomen, thorax, arm, fore-arm, back, and the solei are again commenced with. As soon as these rubbings are begun, a mouth-wash of alum, chlorate of potash, &c., is ordered for the patient, who is instructed to carry it in his pocket, and use it regularly every half-hour; and thus it happens that an enormous amount of mercury can be used without any symptoms of salivation or mercurial stomatitis appearing; but should these appear, the inunctions are stopped for a day or two, the bath being continued, and the rubbings recommenced as the mouth symptoms disappear. I am bound to admit that the result of this treatment is often marvellously successful, and one case I saw specially serves to illustrate this point. A gentleman I met there had been nearly twelve months under the care of two celebrated London surgeons, who are quoted as authorities on syphilis. He had syphilitic psoriasis badly—so badly, that when his London surgeon ordered him to Aachen, as a case almost incurable and most inveterate, he was unable to use either of his hands, or flex his fingers. After fifteen baths and rubbings, the psoriasis entirely disappeared from his hands; he was ordered to have thirty more baths and rubbings, but business called him home to London, where I introduced him to an English medical man, who practises the Aachen system of cure, as far as it is possible to do so without the baths of the genuine mineral water, which latter is largely imported to this country to be drunk. Cases are cured at Aachen which have in England defied mercury taken internally, mercurial fumigations, iodide of potassium, arsenic, iron, and every remedy usually used. The waters are very useful in some cases of skin disease; and Dr. Schumaker ii., who greatly distinguished himself as a military surgeon during the Franco-Prussian war, and is now one of the leading practitioners in Aachen, and under whose kind, attentive, and skilful care I placed myself when there, has written (in English) some very clever articles, illustrative not only of this point, but of others of almost equal interest.

I think it but fair to those who may be sending patients, or going to Aachen, not to close these remarks without some allusion to a few points which struck me when there as a foreigner, and an English medical man. Aachen is a large commercial centre; the visitors, the waters, the baths, the medical details, all play second or third fiddle to the local industries; and there is a section of the residents who are absolutely opposed to visitors suffering from syphilis and skin disease coming to the city:—thus it happens that any enterprise calculated to attract invalids there is always more or less dwarfed. There is neither a club, casino, pump-room, or fashionable indoor place of meeting for visitors. The town band performs, during the summer, in some small public gardens, to which admission is obtained by subscription.

The hotels barely come within the limits of first-class, and the German style of living is hardly in accordance with English tastes. I stayed at the best hotel; the large doors, through which carriages drove into the court behind, led directly into the building, and, as they were nearly always open, the house was somewhat draughty and cold; whilst the absence of fires in, and the tiled uncarpeted floors of, the dining and smoking-rooms were not calculated to assist in curing rheumatism. The dwarfed German beds placed against the bedroom walls are uncomfortable for a restless invalid in pain. The meals were neither liberal, luxurious, nor varied; but the cost of living is fairly moderate, although wines are very dear. The temperature in the shade varies in spring as much as thirty degrees Fahrenheit during the twenty-four hours. Ten different nationalities were represented at my hotel table d'hôte, and it was amusing to notice that the conversation at dinner, after taking turns of a domestic, social, and political kind, invariably, with the *post-prandial* coffee and cigarette, resolved itself into the pathology, cure, and treatment of syphilis, the topic of the day with the majority of the diners. The Kaiserbad is a large building, within which the mineral spring rises; the upper part of it accommodates visitors taking the baths and waters, who can obtain their breakfast and luncheon on the premises, but who have either to go to a hotel to dine, or pay heavily for that meal to be provided in the house. It is fairly well arranged; but the temperature indoors was never up to 60° all the time I was there. The baths in the basement are about thirty in number, and are arranged in rows in a large tiled hall, which, from the heat of the mineral waters, is at a temperature of 74°. They are large, each one holding over three hundred gallons of water; but it is a difficult matter to sit up to the neck in water of a high specific gravity, and some arrangement to support the bather, to enable him to float on the water, would be a great addition; and I found that the cold stone upon which my occiput rested frequently gave me a stiff neck. The dressing-rooms have tiled floors, and are scantily furnished; a couch, on which to rest after taking a hot bath, would be welcomed in them. A *peignoir* (bathing-gown) is provided, and one small towel, both of which smell so offensively of potash soap, that I advise each bather to bring and use his own, washed at home. The bath attendants are most attentive and obliging, and keep the baths clean and in good order. The environs of Aachen are for the most part devoid of interest; and, although "a winter cure" is spoken of by the hotel proprietors, I should imagine that the low temperature, coupled with the paucity of visitors and the dull monotomy of a life indoors, would all combine to depress any invalid on the one hand, and retard his cure on the other. I am, on the whole, bound to admit that I am a firm believer in the virtues of the mineral waters of Aachen, and should never hesitate to send patients to use them during the summer months; but should anyone thinking of trying them during the winter, ask my advice before journeying there, I should feel it my duty to enlarge upon some of the details (both for and against the place) which this paper contains.

